# Intervention Versus Medical Management of Pulmonary Embolism

**DOI:** 10.14797/mdcvj.1351

**Published:** 2024-05-16

**Authors:** Thomas M. Loh

**Affiliations:** 1 Houston Methodist DeBakey Heart & Vascular Center, Houston Methodist Hospital, Houston, Texas, US; 2 Houston Methodist, The Woodlands, Texas, US

**Keywords:** pulmonary embolism, cor pulmonale, risk stratification

## Abstract

With a multitude of options for pulmonary embolism management, we review the most common diagnostic tools available for assessing risk as well as how each broad risk category is typically treated. Right heart dysfunction is the cornerstone for triage of these patients and should be the focus for decision-making, especially in challenging patients. We aim to provide a modern, clinical perspective for PE management in light of the multitude of intervention options.

## Introduction

Anticoagulation has long been the mainstay therapy for treatment of acute pulmonary embolism. Multiple pharmacological and interventional adjuncts have been developed. Many of these adjuncts have been shown to benefit patients with pulmonary embolism (PE) in limited prospective and retrospective studies, but an all-encompassing algorithm has remained elusive secondary to difficulties in studying this patient population. One of the most challenging aspects of caring for patients with PE is the diversity of presentations; variability in acuity, severity, and patient clinical background can make decision-making difficult. However, currently published guidelines are comprised of weak recommendations based on limited data. This allows for some latitude when designing treatment programs but also results in slow adoption of new treatment options by many programs. Despite clinicians receiving periodic updates to these guidelines, little has changed on an interventional front. Acquiring large randomized controlled trials is difficult at baseline but especially challenging when studying emergent pathologies with heterogeneous presentations.^1,2^

Pulmonary embolism triage is the cornerstone for good management, although delineating how sick a particular patient is has been challenging. The Pulmonary Embolism Severity Index (PESI) was developed based on more than 15,000 patients and has been validated externally in multiple other subpopulations. While useful in determining patients at risk for poor outcomes, it is difficult to differentiate between modifiable and nonmodifiable risk factors.^3,4^ We therefore use PESI as a general screening tool in our practice and for prognostication, but rarely does it play a large role in determining treatment modality.

## Right Heart Dysfunction

Cardiovascular collapse in acute PE is caused by cor pulmonale or dilation of the right ventricle secondary to increased pulmonary pressure.^5^ For this reason, despite its name, PE is better thought of as a cardiac rather than respiratory pathology. Thus, the most salient factor in PE triage is heart dysfunction. Acute right heart failure can be assessed by history, physical examination, electrocardiogram (ECG), computed tomography (CT), echocardiogram (echo), and cardiac biomarkers.^6^ Often, these provide little value individually, but when combined together, they can often paint a striking picture of the patient’s current cardiovascular status.

History and physical examination have been used in the diagnosis and assessment of PE severity since the late 1800s.^7,8^ A history of present illness of a patient with cor pulmonale will often include shortness of breath, chest pain, cyanosis, and syncope. Physical examination findings include jugular venous distension, tricuspid regurgitation murmur, and accentuated pulmonic heart sounds.^9^ In an era of easily accessible modern imaging, the role of history and physical examination is often downplayed in favor of the imaging studies. This is problematic for two reasons: First, imaging, while often rapid, can sometimes delay care for patients; second, history and physical examination can sometimes denote severe right heart dysfunction, even in the absence of other indicators allowing for rapid assessment and triage. In our high-volume center’s experience, a history of syncope and new-onset atrial fibrillation with rapid ventricular response have a high correlation with clinical decline.

Electrocardiographic findings during PE include T-wave inversion in leads V1-V4, new right bundle-branch block, and the “classic” S1Q3T3 pattern (S wave in lead 1, Q wave in lead 3, and an inverted T wave in lead 3). While useful on a board test, the most common abnormality on ECG is sinus tachycardia, and new-onset atrial fibrillation with rapid ventricular response, while uncommon, denotes a significant amount of myocardial strain.^10,11^

The wide access to and availability of high-quality CT imaging have made it the most common modality to diagnose PE. Beyond the ability to diagnose the presence of pulmonary emboli, CT scans often contain a plethora of information relevant to heart dysfunction. Despite echocardiograms being considered the gold standard for heart function, CT scans are increasingly relied upon secondary to their ubiquity and lack of operator dependance. Signs of right heart dysfunction on CT include right ventricular enlargement compared to the left ventricle (RV/LV ratio), caval and hepatic reflux of contrast, and abnormal positioning of the interventricular septum.^12,13,14^ The RV/LV ratio is the most utilized metric owing to its objective nature and reproducibility. To obtain the RV/LV ratio, the CT scan is assessed in the axial view and the diameter of the RV and the LV are measured perpendicular to the septum. The largest measurements for each chamber are then compared, regardless of which level the measurement was acquired. A ratio > 0.9 is indicative of right heart dysfunction.^15^ When clear signs of cardiac dysfunction are present on CT scan, triage is often made simple.

Echocardiogram has long been considered the standard bearer for assessment of right heart dysfunction. A multitude of studies have validated its assessment, and when triage or clinical decision-making is in doubt, echo is the study of choice. Despite this, availability and duration of the study can hinder its use in critically ill patients with PE. Findings suggestive of right heart dysfunction include RV dilatation, decreased RV systolic function, increase in pulmonary artery systolic pressure, impaired LV diastolic filling, abnormal interventricular septum, and intracardiac thrombus.^16,17^

Cardiac biomarkers including troponins and B-type natriuretic peptide (BNP) are secreted by myocardium when under strain and are detected in the bloodstream rapidly after cardiac insult. While nonspecific for PE, they represent acute nonischemic myocardial injury in the presence of a large PE.^18,19,20^

**Figure 1:**
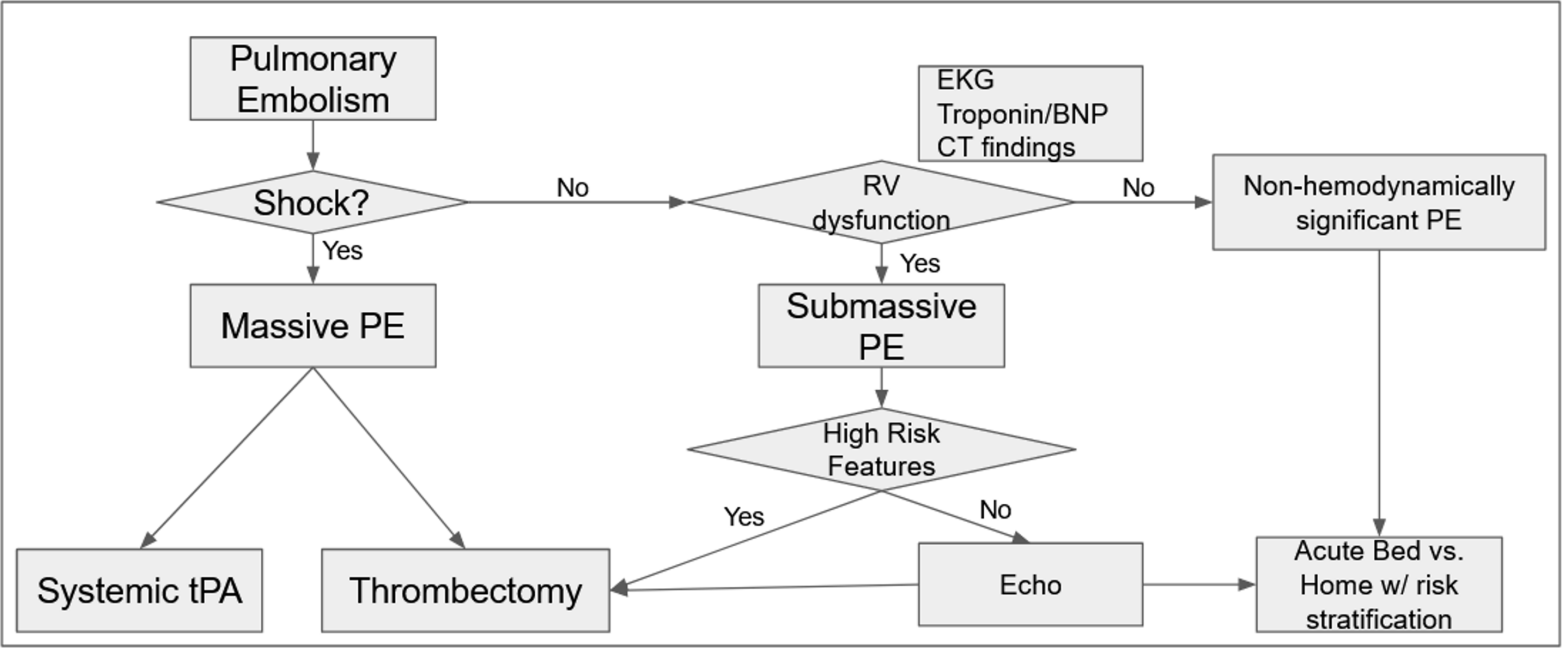
Sample algorithm for pulmonary embolism triage. EKG: electrocardiogram; BNP: brain natriuretic peptide; CT: computed tomography; PE: pulmonary embolism; tPA: tissue plasminogen activator

Each of these modalities has their place in the triage and risk stratification of patients with PE. However, creating a single algorithm for all facilities is difficult owing to differing resource availability. At our facility, like many others, high-quality CT scans are how most pulmonary emboli are diagnosed, and echocardiograms are sometimes difficult to obtain quickly. We find in our hospital that beyond the CT scan, vitals, cardiac biomarkers and the patient’s history of present illness offer the most reliable way to determine right heart status (see proposed algorithm, Figure 1). We utilize echocardiogram for specific patients with confusing or inconsistent signs and symptoms, and we advocate an individualized facility approach to this dependent on local resources.

## Risk and Benefit Assessment

Triage for PE mandates an assessment of the risk of pathology, with high morbidity and mortality, against the risks and benefits of various medical and interventional treatment strategies. It is very important to recognize that these potential risks and benefits of intervention are not static and are often specific to the facility. Individual facility familiarity with particular techniques will often be more important than the type of interventional technique used. Often the single most important initial improvement in PE care at a particular center is the provider’s mandatory evaluation of the patient, which can provide medical and interventional therapy as well as regular follow-up.

## Pulmonary Embolism Risk

Pulmonary emboli are divided into four categories of risk: low, intermediate-low, intermediate-high, and high risk. These categories are based on a patient’s risk of cardiovascular collapse. Patients with no signs or symptoms of right heart dysfunction are considered to be low risk. If a patient has signs or symptoms of right heart dysfunction but is hemodynamically stable, the patient is considered to be at intermediate risk. If a patient has both biomarker and imaging signs of right heart dysfunction, they are considered to be intermediate high risk. If only one risk is present, then the patient is considered to be intermediate low risk. Lastly, patients with a systolic blood pressure under 90 mm Hg or requiring pressor support are considered to be high risk or to have massive pulmonary emboli. There is significant interest in establishing broader definitions for high-risk pulmonary emboli since patients who would otherwise be considered to be in cardiogenic shock do not meet criteria for high-risk PE.^21,22^

In reality, patients exist on a dynamic spectrum and often slide from one category to another over time. Rapid changes should be anticipated in patients with high-risk features such as new-onset atrial fibrillation, a history of syncope related to the PE, or clot in transit. It is prudent to recognize that these high-risk features may tip the calculus of risk versus benefit in individual patients.

### Low-Risk Pulmonary Embolism

Anticoagulation remains the treatment of choice for low-risk PE among published guidelines. There is a lack of recent discussion or new literature aimed at changing this paradigm.^1,2^ While little new literature exists for more advanced reperfusion techniques in this patient population, the lack of demonstrated benefit for these patients greatly outweighs the potential increased risk, especially for strategies involving thrombolysis.^23^

### Intermediate-Risk Pulmonary Embolism (Submassive Pulmonary Embolism)

Significant disagreement exists over the management of intermediate-risk pulmonary emboli. Current guidelines provide for advanced reperfusion options when there is clinical deterioration of the patient on anticoagulation and limited other situations. These are based mostly on studies with thrombolytics and do not account for the ever-widening availability of percutaneous mechanical thrombectomy.^1,2^ Limited data has been published about the efficacy of intervention in intermediate-risk PE. Catheter-directed thrombolysis and percutaneous mechanical thrombectomy have demonstrated improvement of right heart function at 24 to 48 hours, but long-term change in mortality has not been established.^24,25,26^

Guidelines in this area are likely to change considerably in the near future as this is the most studied area of PE care. There is a clear segment of this risk group that will benefit from advanced reperfusion interventions, and it is highlighted in the current guideline recommendations; however, until more data is available, specific recommendations and guidelines will be limited. At our facility, we use a highly individualized approach to intermediate-risk pulmonary emboli. The focus of this approach is current right heart function and variables that make the patient high risk for sudden decline.

### High Risk Pulmonary Embolism (Massive Pulmonary Embolism)

It is nearly universally agreed that, if possible, patients with high-risk PE should undergo a reperfusion strategy beyond anticoagulation.^1,2^ With the advent of multiple new percutaneous thrombectomy devices and the ability to rapidly offload the right heart faster than can be achieved with thrombolysis and open pulmonary embolectomy, there is significant interest in treating these patients aggressively. However, this has historically been a very challenging cohort of patients to study. There is a lack of data assessing the merits of different reperfusion strategies owing in no small part to these patients often being excluded from clinical trials as well as their relative low incidence compared to intermediate-risk patients.

A recent publication on large bore mechanical thrombectomy specifically in high-risk patients demonstrated superior results compared to both an historical context and contemporary patients who underwent other reperfusion strategies.^27^ An obvious limitation of this data is its nonrandomized nature, which was expected and accepted when the trial was designed. There appears to be the expected selection bias from a prospectively maintained registry.^28^ Given the lack of data and difficulty with studying this patient population, we recommend highly individualized assessment of these patients based on the available local resources. As the risk profile of interventions changes, it is likely that advanced reperfusion strategies will significantly improve outcomes in these patients.

## Key Points

- Pulmonary embolism risk is determined by right heart dysfunction.
- History and physical exam remain crucial for early pulmonary embolism diagnosis despite advances in imaging.
- Triage algorithms should be tailored to specific facilities based on available expertise and resources.

## CME Credit Opportunity

Houston Methodist is accredited by the Accreditation Council for Continuing Medical Education (ACCME) to provide continuing medical education for physicians.

Houston Methodist designates this Journal-based CME activity for a maximum of *1 AMA PRA Category 1 Credit*™. Physicians should claim only the credit commensurate with the extent of their participation in the activity.

Click to earn CME credit: learn.houstonmethodist.org/MDCVJ-20.3.
